# Establishing the soundness of administrative spatial units for operationalising the active living potential of residential environments: an exemplar for designing optimal zones

**DOI:** 10.1186/1476-072X-7-43

**Published:** 2008-07-31

**Authors:** Mylène Riva, Philippe Apparicio, Lise Gauvin, Jean-Marc Brodeur

**Affiliations:** 1 Département de médecine sociale et préventive, Université de Montréal, Montréal, Canada; 2 GRIS, Groupe de recherche interdisciplinaire en santé, Université de Montréal, Montréal, Canada; 3 Centre de recherche Léa-Roback sur les inégalités sociales de santé de Montréal, Université de Montréal, Montréal, Canada; 4 Institut National de la Recherche Scientifique – Urbanisation, Culture et Société, Montréal, Canada; 5 Institute of Hazard and Risk Research, Department of Geography, Durham University, UK; 6 Analyse et évaluation des interventions en santé, AnÉIS, Université de Montréal, Montréal, Canada

## Abstract

**Background:**

In health and place research, definitions of areas, area characteristics, and health outcomes should ideally be coherent with one another. Yet current approaches for delimiting areas mostly rely on spatial units "of convenience" such as census tracts. These areas may be homogeneous along socioeconomic conditions but heterogeneous along other environmental characteristics. This heterogeneity can lead to biased measurement of environment characteristics and misestimation of area effects on health. The objective of this study was to assess the soundness of census tracts as units of analysis for measuring the active living potential of environments, hypothesised to be associated with walking.

**Results:**

Starting with data at the smallest census area level available, zones homogeneous along three indicators of active living potential, i.e. population density, land use mix, and accessibility to services were designed. Delimitation of zones ensued from statistical clustering of the smallest areas into seven clusters or "types of environment". Mapping of clusters into a GIS led to the delineation of 898 zones characterised by one of seven types of environment, corresponding to different levels of active living potential. Homogeneity of census tracts along indicators of active living potential varied. A greater proportion (83%) of variation in accessibility to services was attributable to differences between census tracts suggesting within-tract homogeneity along this variable. However, census tracts were heterogeneous with respect to population density and land use mix where a greater proportion of the variation was attributable to within-tract differences. About 55% of tracts were characterised by a combination of three or more "types of environment" suggesting substantial within-tract heterogeneity in the active living potential of environments.

**Conclusion:**

Soundness of census tracts for measuring active living potential may be limited. Measuring active living potential with error may lead to misestimation of associations with walking, therefore limiting the correctness of inference about area effects on walking. Future studies should aim to determine homogeneity of spatial units "of convenience" along environment characteristics of interest prior to examining their association with health. Further evidence is needed to assess the extent of this methodological issue with other indicators of environment context relevant to other health indicators.

## Background

Residential areas are proximal to everyday life and are therefore likely to influence health of local populations through the possibility they provide for leading healthy lives [1,2]. An accumulating body of research shows evidence for variation in health across residential areas and the significance of area context for explaining this variation, independently of the characteristics of individuals [3-5].

Different scales, or spatial units, may be relevant to specific contextual conditions and to specific heath outcomes [6,7], as illustrated by studies reporting varying strength and magnitude of area effects on health according to the operational definition of areas [8-15] or to contextual conditions [16-19]. Nonetheless current approaches for delimiting areas mostly rely on spatial units "of convenience" such as census tracts, boroughs, or wards [3,5]. These spatial units are certainly useful because they can easily be linked to data from censuses and other surveys that can be used for measuring contextual conditions. Also, they are often designed to be homogeneous along socioeconomic conditions of populations, thus being appropriate spatial units to operationalise the socioeconomic context of areas [20] (this may not hold for other administrative units, e.g. postal code areas which are design for postal delivery purposes and may be very heterogeneous in terms of population composition). However it is to be considered that through time, the composition of the units may change leading to modification of the socioeconomic conditions which may become more heterogeneous.

Yet, other contextual dimensions relevant for health may not be optimally defined within administrative spatial units. For example, conduciveness of areas to physical activity or geographic accessibility to health services may operate on different scales than socioeconomic factors. Operationalising relevant spatial units for studying area effects on health remains a conceptual and methodological challenge [4,5,7,21-26] giving rise to issues of validity and soundness of areal units as units of analysis [27].

### Operationalising small areas: issues of validity and soundness units of analysis

Construct validity refers to whether or not the measurement instrument operationalises the concept of interest. In area effects on health research, construct validity is a matter of establishing 1) the soundness of units of analysis, i.e., whether or not area boundaries are aetiologically meaningful for studying the association between area characteristics and a given health indicator, and 2) whether or not data constitute appropriate operationalisations of exposure variables, i.e. the characteristics of areas [27]. Ideally, definitions of areas, the characteristics of these areas, and the health outcome(s) being studied should be coherent with one another [7].

Measures of area characteristics derived from population censuses and other surveys, e.g. socioeconomic position, although easily accessible, provide only partial information on the context of areas and may in fact be endogenous to the composition of the areas as they are determined by individual characteristics of residents [28]. Collecting and measuring "true" or "integral" area data, i.e. data only measurable at the area level through procedures such as ecometrics and spatial analyses has been underscored as critical for measuring unbiased area-level variables [2,7,28,29]. Likewise, defining aetiologically meaningful areas in coherence with the specific purposes of the study, either in terms of health outcomes, characteristics of environment, or associations between the two [23,28,30,31] is important for understanding the significance of residential areas for health. Measurement errors can result if the spatial patterning of environmental characteristics does not correspond to the spatial units chosen for operationalising areas and their context [27].

Defining relevant geographic areas becomes salient in light of the modifiable areal unit problem, i.e. the fact that analytical results are sensitive to the definition of spatial units at which data are aggregated [32,33]. In other words, area effects may be observed only at certain scales, i.e. scales at which data are collected and aggregated and may vary or be absent when observed at other scales. Imposing arbitrary spatial units on a continuous spatial process, e.g. characteristics of environments, may lead to the delineation of artificial spatial patterns. In such cases, environment characteristics may be measured with error. As a result the internal validity of the study, i.e. whether or not observed associations are unbiased, may be threatened.

In addition, as per spatial autocorrelation, areas will share similar contextual conditions as a function of their proximity in space [34]. By using spatial units of convenience, it is assumed that contextual conditions within one area are different and influence health independently of conditions in neighbouring areas [4,5,21,22,24-26,35], when in fact these conditions are clustered in space. Furthermore, for any area effects to be detected there must be variation in the exposure being studied [36]. Yet the variation of environment characteristics may be smoothed out by the definition of area units used to measure them. For example, if spatial units encompass environments that are both conducive to walking and others that are less so, averaging values of conduciveness over census tracts could potentially lead to mismeasurment of exposures. Within area homogeneity along the contextual conditions under examination is thus required for minimising measurement error. Correspondingly, for inferring about area effects on health, between area differences must be maximised: if data are collected in contiguous and heterogeneous areas, variations in both characteristics of environments and health outcomes, and their association, may be misestimated. As area effects on health have been observed to be stronger in more homogeneous areas [37,38], homogeneity of areas may thus influence the estimation of area effects and therefore the validity of conclusion.

**Figure 1:**
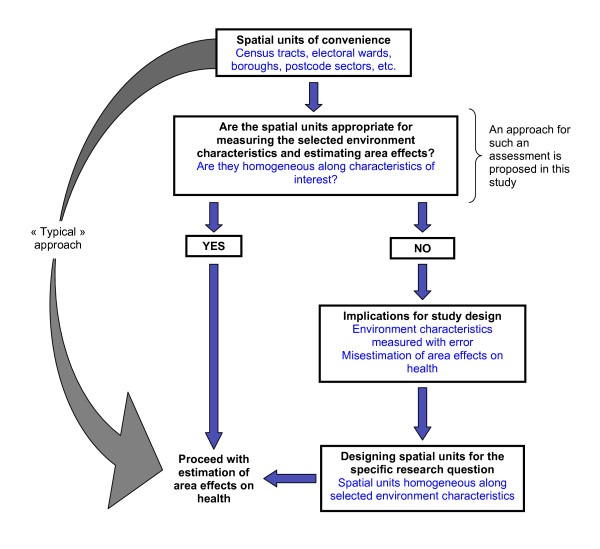
**Template for deciding upon the soundness of "spatial units of convenience" to operationally define small area units of analysis**.

In Figure 1, we propose a template that could be useful for establishing the soundness of spatial units "of convenience" to operationally define areas for specific research questions. For example, the template could be used to guide the decision as to whether or not census tracts are the most appropriate spatial units of analysis for measuring associations between area-level socioeconomic position (SEP) and obesity. That is, if they allow for measuring indicators of SEP without bias (and ultimately for estimating non-biased association with health outcomes) by showing homogeneity in the distribution of indicators and optimising their spatial patterning. In the methods section, we propose an approach for achieving this end. Intuitively, it can be expected that census tracts are appropriate units for undertaking such a study as they are, as mentioned above, initially designed to be homogeneous along socioeconomic conditions. But across time, the socioeconomic composition of census tracts may change as people migrate in and out of areas, potentially introducing heterogeneity in the socioeconomic make-up of the area. This could result in a "dilution" of the true level of deprivation. Averaging indicators of SEP over census tracts thus may mask "pockets" of poverty. The exercise of establishing the soundness of census tracts as units of analysis would be important here, as it would allow to measure with less error indicators of SEP and their association with health outcomes. In multilevel studies, mismeasurement of environment characteristics may influence the strength of the observed association between environment characteristics and health indicators [39]. As such, associations may not be detected or may be spurious, therefore limiting the precision of research findings for informing public health and public policy actions to tackle social and geographical inequalities in health.

Establishing the soundness of spatial units of analysis chosen for operationalising area boundaries and measuring area context is an important methodological consideration, but it is often overlooked. Alternatively, designing spatial units of analysis maximising homogeneity of selected environment characteristics may prove to be a viable strategy for advancing the understanding of processes linking place to health [7].

## Objectives

The aim of this investigation is to assess the soundness of census tracts as units of analysis for studying associations between a specific exposure and a specific health outcome, namely the active living potential of residential environments and walking behaviours. Active living potential refers to the conditions of areas that encourage the likelihood of integrating physical activity into daily routines [29]. Census tracts were selected as spatial units "of convenience" because of extensive use of this spatial unit of analysis in current research on health and place [4,5]. In Canada, census tracts are small and relatively stable geographic areas with populations ranging in size between 2500 and 8000 inhabitants; at the time of their creation, census tracts were homogeneous in terms of socioeconomic characteristics, e.g. economic status and social living conditions [40].

To establish the soundness of census tracts as a unit of analysis, we developed and tested a comprehensible method for designing optimal and homogeneous spatial units espousing the spatial distribution of selected environment characteristics linked to the concept of density of destinations that is the physical and social characteristics of residential areas related to land use pattern [29]. Three indicators were used to operationalise the construct of active living potential: population density, land use mix, and geographic accessibility to proximity services. The specific objectives of the study are to examine whether or not: 1) census tracts are homogeneous units of analysis along indicators of active living potential; 2) active living potential and socioeconomic indicators follow a similar spatial distribution; and 3) census tracts encompass smaller areas with different (or similar) levels of active living potential.

Active living potential was chosen because of increasing research reporting associations between this environmental construct and walking [19,41-53], an important public health indicator [54-56]. This choice was also motivated by availability of spatial datasets allowing for the operationalisation of integral measures of land use mix and geographic accessibility to services in geographical information systems, and by the availability of individual-level data on walking behaviours (to be examined in future analyses).

## Methods

The methodology section includes two parts. First, we present criteria and methods for designing homogeneous areas (henceforth designated as "zones"). Second, we present analyses undertaken to assess the soundness of census tracts as units of analysis for measuring the active living potential of residential areas.

### Designing optimal, homogeneous zones

Zone design refers to the placement of areal unit boundaries [9]. It can be achieved discursively (manually) by grouping basic spatial units into larger ones [57-59], by combining social, statistical, and spatial analysis methods [60,61], and automatically through computationally intensive automated zoning software [9,15,37,62-65].

Three criteria guided the choice of the method for zone design. First, we wanted to design zones based on the spatial distribution of environmental characteristics related to active living potential, namely population density, land use mix, and geographic accessibility to selected proximity services. We had no requirement regarding population and area sizes as zones were defined on the basis of the spatial distribution of these characteristics. Second, the method for zone design had to be optimal, i.e. to maximize variation between zones and to minimize variation within zones in the selected characteristics. In other words, the aim was to design zones that were internally homogeneous on the three indicators of active living potential, but different (heterogeneous) amongst themselves. Finally, we wanted a method that was rigorous but comprehensible and easy to implement. We opted for an approach that combined a statistical classification method, *K*-means clustering, to mapping applications in geographic information system. This three-step approach is described in greater details in the following sections.

#### Step 1: Measuring environment characteristics at the smallest area level

The study area is the Island of Montreal, Canada, an urban centre with 1 812 723 residents. As of January 2006, on the Island of Montreal, there are 15 municipalities, in addition to the municipality of Montreal which includes 19 boroughs [66]. The Island of Montreal is further divided into 521 census tracts and 3222 dissemination areas. Dissemination areas (DAs) were used as basic spatial units for designing zones because they are the smallest standard geographic areas for which Canadian census data are available (population size between 400 and 700 residents) [40]. On the Island of Montreal, their average size is 0.15 km^2 ^(ranging between 364 m^2 ^and 18 km^2^) with an average population of 562 individuals (ranging between 44 and 2138 residents). DA values for population density, land use mix, and accessibility to services were computed in a geographical information system (ArcGIS 9.2) [67].

*Population density *refers to number of individuals per unit area. It was computed by dividing the total number of residents of a DA by its area size (km^2^) [68].

*Land use mix *relates to the diversity or variety of land uses within an area. It was computed using an entropy index [47,69,70] which measures the homogeneity or diversity of land uses within a spatial unit. The index is defined as follow:

(1) $$E_{j}=-\sum_{i=1}^{n}\left[\left(A_{ij}/D_{j}\right)\ln\left(A_{ij}/D_{j}\right)\right]/\ln n$$

Where A_*ij *_is the surface area of land use *i *in dissemination area *j*, D_*j *_is the surface area of dissemination area *j*, and *n *is the total number of possible land uses which in the current case corresponds to 16, the number of different land uses characterising the Island of Montreal [71]. The index values range between 0 and 1, where 1 corresponds to a highly mixed area, and 0 to a homogeneous area, that is an area characterised by only one type of land use (e.g. low density housing). This index has been used in many studies to measure land use mix [47,72].

*Geographic accessibility to proximity services *refers to geographic distance to or from destinations, here to supermarkets, pharmacies, banks, and libraries. These services were selected because they are most likely to be used on a regular basis, conveying the idea of proximity services potentially accessible through walking. There are many measures of geographic accessibility [73,74]. In this study, geographic accessibility was defined in terms of the number of the selected services within an area, conferring the notion of the offer of services provided by the immediate surroundings. Supermarkets, pharmacies, banks, and libraries were geocoded at the parcel level [75]. In order to minimise aggregation errors [73,76], accessibility was measured by computing distances of services located within a one kilometre (network distance) radius [77] from the centroid of census blocks (n = 14 527) comprised within any one DA; the distances were than averaged and weighted by the total population of each census blocks.

Characterisation of DAs along the three indicators resulted in a sample of 3206 DAs. Measures of land use mix and accessibility to services were normally distributed; population density was normalized using a LOG10 transformation [78]. Population density was significantly and positively correlated to accessibility to services (r = 0.45, p < 0.001), and negatively to land use mix (r = -0.32, p < 0.001). Land use mix and accessibility to services were not significantly correlated (r = 0.03, p > 0.500). Prior to cluster analyses, these variables were standardized to a mean of 0 and a standard deviation of 1, higher values representing greater levels of population density, land use mix, and accessibility to services.

#### Step 2: Classifying smallest areas into clusters, e.g. "types of environments", using K-means clustering

*K*-means statistical clustering techniques using SAS (version 9.1) for Windows [79] was applied to classify DAs into *k *number of optimal clusters homogeneous in terms of active living potential. In social sciences, notably in geography, *K*-means is largely employed to classify areas (e.g. geodemographics [80]). The method uses an allocation/re-allocation algorithm to optimally reassign objects, here DAs, to the nearest cluster centroid [81-83]. The goal is to maximize between cluster variations and to minimize within cluster variations. The aim of this second step was to group DAs with similar values of population density, land use mix, and accessibility to services into *k *types of environments that are internally homogeneous but different among them. These types of environments correspond to different levels of active living potential. For *K*-means clustering, the number of clusters (*k*) must be determined at the onset of analyses; as we had no a priori for such number, we conducted analyses for *k *= 4 to *k *= 20.

#### Step 3: Mapping the clusters to create optimal and homogeneous zones

In a final step, the *k *types of environments were imported into ArcGIS 9.2 and mapped out. This lead to the delineation of *n *homogeneous zones i.e., units of analysis, characterised by one of *k *active living potential.

### Statistical analyses: Assessing the soundness of census tracts as units of analysis for operationalising active living potential

The soundness of census tracts for operationalising indicators of active living potential was assessed through three series of analyses.

First, to assess the homogeneity of census tracts, variation in indicators of active living potential was estimated and decomposed between and within areas. Population density, land use mix, and accessibility of services were measured continuously at the DAs level (level 1: n = 3206). In separate two-level multilevel models, DAs were nested into zones (n = 898) and into census tracts (n = 506 with valid population and socioeconomic data). Between-area variation in indicators of active living potential was estimated using the intraclass correlation coefficient (ICC) from unconditional (null) multilevel models using HLM software Version 6.04 [84]. The ICC indicates the proportion of variation in a dependent variable that is attributable to differences between area units. Greater ICC values indicate that variation of a variable is greater between units than within, i.e. units are different among them but internally homogeneous. Using the same analytical approach, homogeneity of zones and census tracts along indicators of socioeconomic position was assessed and compared. DA-level data on the proportion of low-income households, of people with less than high school education, and of people with a university degree were obtained from the 2001 Canadian census.

Second, analysis of variance was performed to examine the proportion of variation across zones in socioeconomic variables explained by the *k *types of environment. Indicators of SEP at the DA-level were aggregated (weighted by population) at the zone-level. These analyses were performed to examine whether or not socioeconomic and active living indicators follow a similar spatial distribution as is implicitly assumed when measured within the same area unit of analysis.

Finally, descriptive statistics were employed to assess the extent to which the spatial distribution of the different types of environment coincides with the boundaries of census tracts. These analyses were conducted to examine if census tracts encompassed environments with differing levels of active living potential. The numbers of zones straddling over one or more census tracts, and the number of types of environment encompassed within census tracts were computed. To examine whether or not the spatial distribution of more mixed or more homogeneous census tracts (i.e. the number of types of environments encompassed within census tracts) was structured in space, global values of spatial autocorrelation were computed using Moran *I *with a first-order contiguity matrix [85,86]. Values for Moran *I *vary between -1 and 1, where negative values indicate negative spatial autocorrelation, i.e. neighbouring spatial units have different values, and positive values indicate positive spatial autocorrelation, i.e. neighbouring units have similar values. The covariance in Moran *I *is the covariance over space for neighbouring spatial units, and will not be computed unless two units are contiguous (first order); also, only one variable is considered [85], here the number of types of environments included in census tracts.

## Results

### Description of types of environment and zones

**Figure 2:**
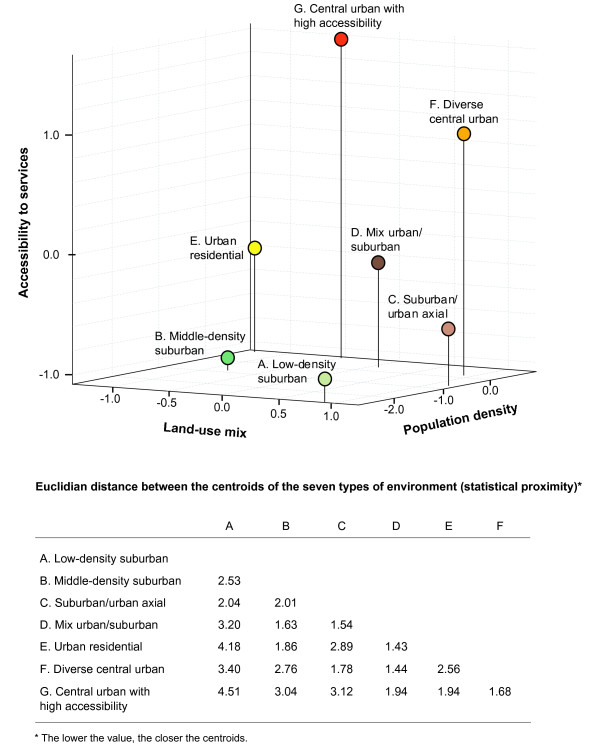
**Statistical proximity of the seven types of environment (clusters)**.

**Figure 3:**
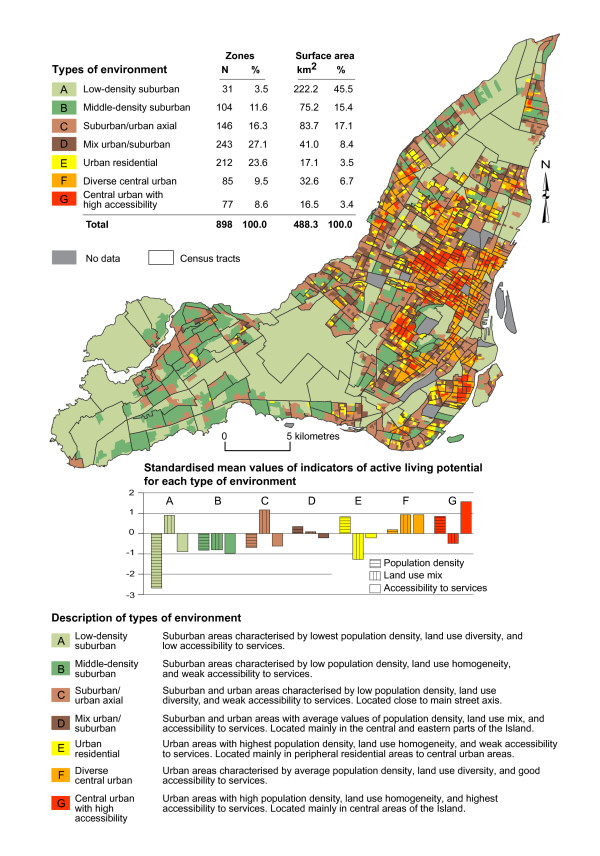
**Description of types of environment (clusters) and zones**.

Figure 2 illustrates results of the *K*-means clustering, which show that the 3206 DAs were optimally classified into 7 clusters or "types of environments" as indicated by peaks [87] in both the Pseudo-F statistic [88] and the Cubic clustering criterion [89]. These clusters explain 72.8% of the total variation in the three indicators of active living potential. Thus, differences among the seven clusters and similarity of DAs comprised within the same cluster, i.e. within-cluster homogeneity, were both maximized. The seven types of environments correspond to seven different levels of active living potential. They encompassed more suburban to more central urban types of environments defined by different values of population density, land use mix, and accessibility to services. The types of environments are described in Figure 2 and Figure 3.

Low-density and mid-density suburban areas are characterised by lower values of population density and accessibility to services. Diverse central urban areas and central urban areas with high accessibility are more densely populated and have greater access to services than any other types of environment. Although population density and accessibility to services follow to some extent an increasing gradient from more suburban to more urban areas, the pattern of land use mix is more complex: there are low values in urban areas and high values in suburban areas. Dissemination areas are designed to be similar in population size (among other characteristics); thus the area size required to reach the set population threshold (i.e. between 400 to 700 residents [40]) will be larger in less densely populated areas and smaller in more urban areas. As a consequence, larger dissemination areas are more likely to encompass different land use than are smaller dissemination areas located in urban areas.

Figure 2 also presents the statistical proximity (Euclidian distance) of the centroids of clusters (cluster mean values), i.e. types of environment, in a three dimensional graph where the axes correspond to the three indicators of active living potential. With respect to their spatial distribution, the types of environment are positively correlated in space indicating that contiguous zones were characterised by similar types of environment.

Mapping of the clusters into the GIS led to the delineation of 898 zones or units of analysis characterised by one of the seven types of environments, i.e. active living potential, as illustrated in Figure 3. Zones are significantly smaller than census tracts, an average of 0.54 km^2 ^(SD = 3.50) compared to 0.96 km^2 ^(SD = 1.98) (t = -2.46; p < 0.05), but the variation of their area size is not statistically different (F = 0.68; p = 0.409). Zones are significantly smaller than census tracts in population size, an average of 1960 (SD = 3867) residents compared to 3554 (SD = 1647) (t = -10.73; p < 0.001), and there is significantly greater variability in population size across zones than across census tracts (F = 11.40; p < 0.01). Zones characterised by more suburban contexts are on average larger and have relatively smaller population counts than urban zones.

### Soundness of census tracts as units of analysis for measuring active living potential

#### Homogeneity of census tracts along active living potential indicators

**Figure 4:**
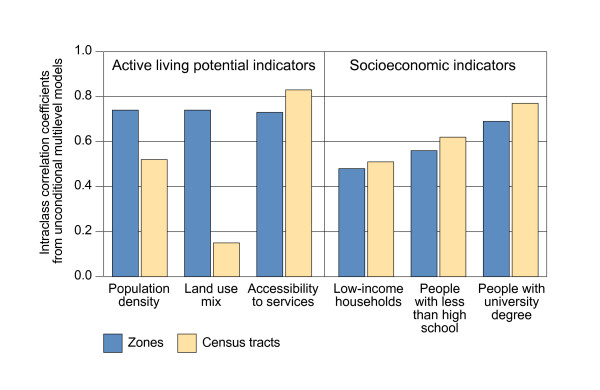
Decomposition of variation in indicators of active living potential and socioeconomic position across zones and across census tracts.

Results of homogeneity of zones and census tracts along active living indicators appear in Figure 4. The variation in indicators is not uniform across census tracts. A greater proportion (83%) of variation in accessibility to services is attributable to differences between census tracts, as indicated by a higher ICC value, suggesting within census tract homogeneity along this indicator. Yet about half of the variation in population density is between census tracts (52%), whereas there is greater variation in land use mix within census tracts (85%), indicating greater heterogeneity of tracts along these indicators. The degree of homogeneity of tracts therefore varies according to the indicator examined. For population density and land-use mix, but not for accessibility of services, variation between zones is greater than variation between census tracts. This shows that the method was successful in designing areas or units of analysis that were more homogeneous than census tracts along dimensions of active living potential.

The degree of homogeneity of census tracts and zones along socioeconomic indicators shows that for the selected variables, variability is larger between census tracts than between zones (Figure 4). Census tracts are relatively homogeneous areas in terms of the socioeconomic environment, especially for proportion of population with a university education.

#### Spatial distribution of active living potential and socioeconomic indicators

Examining variation in socioeconomic indicators across zones shows that they follow a different spatial distribution than that of active living potential indicators. Results of analyses of variance (results not shown) revealed that 15.2% of the variation in the proportion of low-income households was explained by the seven types of environment whereas these proportions were 5.2% for the proportion of people with less than high school and 3.8% for the proportion of people with a university education.

#### Types of environments encompassed within census tracts boundaries

**Figure 5:**
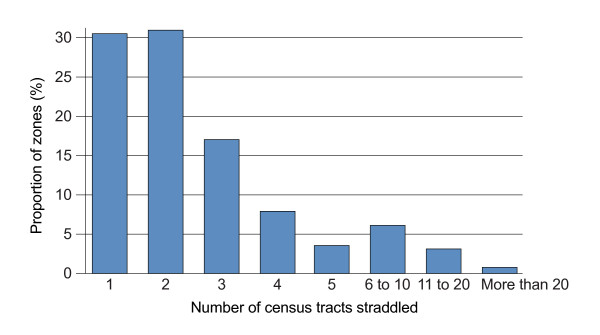
**Proportion of zones straddling different numbers of census tracts across the Island of Montreal**.

Overall, zones are not well contained within census tracts. As shown in Figure 5, only 30.5% of zones are completely located within the boundaries of one census tract. Forty-eight percent of zones straddle two or three census tracts whereas, 21.5% spread over more than four tracts. Correspondingly, there is considerable variability in types of environment within census tracts.

**Figure 6:**
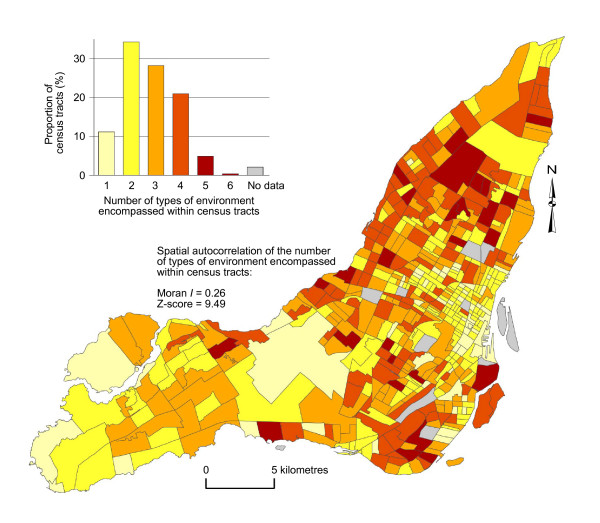
Number of types of environment encompassed within census tract boundaries.

As illustrated in Figure 6, 11.2% of census tracts encompass only one type of environment and 34.3% encompass two types. About 28% of census tracts are characterised by three different types of environment, whereas 26.3% comprise 4 or more different types. Among census tracts encompassing two types of environment (n = 175), about two-thirds (66.3%) comprise types that are statistically similar as indicated by distances between their centroids (two or less distance lag as indicated in the distance matrix in Figure 2; results not shown). For example, census tracts often comprise a combination of low-density suburban and suburban/urban axial zones (26.3%), or a grouping of diverse and high accessibility central urban areas (35.4%). Globally, the number of types of environment encompass within census tracts is positively correlated in space (Moran *I *= 0.26; p < 0.001), suggesting that more homogeneous or more mixed census tracts are often contiguous in space (Figure 6). More heterogeneous census tracts are located mainly on the periphery of central urban areas and in the eastern part of the Island of Montreal, and to a lesser extend in the west-end suburbs.

## Discussion

The objective of this study was to assess the soundness of census tracts as units of analysis, i.e. their degree of homogeneity in terms of the active living potential of residential environments associated with walking. In order to do so, homogeneous zones that optimised the spatial patterning of active living potential indicators hypothesised to be associated with greater involvement in walking, namely population density, land-mix use, and accessibility to services, were successfully designed. This was done through the application of an easy-to-use method combining a classification method called *K*-means clustering with basic mapping applications of geographical information systems. The degree of soundness of census tracts as units of analysis was established through a series of analyses comparing them to the newly-designed zones.

First the distribution of the three active living indicators between and within census tracts was assessed. Although census tracts were homogeneous in terms of accessibility to services, they were less homogenous in population density; for this indicator within and between census tracts variations were about equal. Census tracts were clearly not homogeneous in terms of land use mix as the variability within tracts largely exceeded the variability between tracts. In contrast, census tracts were homogeneous along socioeconomic variables. These results suggest that the spatial patterning of the active living potential of environments do not neatly follow in the delineation of census tracts, which may be more suitable as units of analysis for operationalising socioeconomic contexts.

Then, findings revealed that the spatial distribution of active living and socioeconomic indicators followed different spatial distribution. At the zone-level, types of environment explained a small proportion of variation of socioeconomic variables. This indicates that processes underlying the distribution of active living and SEP indicators, although potentially linked [2,6], operate at different scales and thus require different units of analysis.

In the final set of analyses, within tract variability in terms of what we labelled "types of environment" was examined. This allowed for the assessment of whether or not census tracts encompassed environments that were substantively different among them in terms of their active living potential. Census tracts comprising two different types of environments (34.3%) were not considered necessarily as problematic, given that some types of environment were more similar than others and were often contiguous in space. For example, diverse and high accessibility central urban zones were often contiguous in space and were statistically most similar (as indicated by statistical distances between clusters; Figure 2). However, census tracts comprising three or more types of environment raised concerns; such a situation was observed in more than half of census tracts. These tracts encompass environments that are simultaneously most conducive to walking and others that are least so. Averaging values of conduciveness to walking could potentially lead to significant errors when measuring active living potential at the census tract-level.

The approach for defining areas or units of analysis differs from those involving the definition of strictly "ecologically meaningful" or "natural" neighbourhoods, i.e. neighbourhoods imbued with meaning for residents [21] or as consisting of a group of homes sharing a commonly defined residential area often having name [20]. Defining such units of analysis is important when the notion of commonly shared territory is related to the contextual condition of interest, for example social capital or collective efficacy [7,27]; this notion is not conjured up by active living potential. Designing zones based on the spatial distribution of active living indicators empirically linked to greater involvement in walking leads to the definition of areas that are more appropriate units of analysis and increases the internal validity of study design examining the environmental determinants of walking.

Future studies are needed to assess the impact of the choice of other environmental characteristics for designing zones relevant to other health indicators, and to other geographical areas. For example, areas relevant for studying the social and environmental determinants of overweight and obesity may be delimited according to the distribution of active living variables and food provision (accessibility of both healthy and non-healthy food). For studying mental health outcomes, social dimensions of area context such as social support and opportunities for social participation, may be more relevant. It is to be expected that designing zones using other indicators of contextual conditions associated with other health outcomes will lead to different spatial configuration of area units of analysis.

Homogeneous zones are designed with the aim of optimising the study of a phenomenon or for the purpose of uncovering the aetiology underlying associations between area context and health. As such, the configuration of zones should not be viewed as other "spaces" of actions for public health and policy interventions. Rather, they may be useful for informing on viable interventions and policy strategies that may be health promoting.

### Limitations

Results of this study should be considered in light of some limitations. First, there is a seven year time lag (2000 to 2006) between the dates of creation of the different datasets used to characterise dissemination areas in terms of their active living potential and socioeconomic position. Although changes in the built environment may have taken place during this period, the speed at which changes occur is not well documented; however over a seven-year period, changes in the built environment can be expected to be modest.

Other indicators of active living potential could be examined in designing homogeneous areas, such as street connectivity, safety, and accessibility to other services or resources such as parks. In this study, the measurement of land use mix was dependent on the size of disseminations areas which are defined in part by a population size threshold: because of lower population density in suburban areas, DAs are likely to span a greater territory and therefore encompass more types of land use. Other scales for measuring land use mix could be considered [47].

## Conclusion

For studies concerned with the social and environmental determinants of health and more specifically of physical activity, results of this study have several implications. Delimiting areas is a key conceptual and methodological challenge in research on health and place. In this paper, we developed an easy-to-use method for establishing homogeneous units of analysis in terms of specific environmental characteristics hypothesised to be linked to a specific health indicator. The focus was on active living potential of areas and walking behaviours. Using these homogeneous zones as comparison, the objective was to assess the soundness of spatial units "of convenience", i.e. census tracts, to operationalise contexts for which they were not purposely developed. The methods developed in this study add to the growing literature on alternative ways to conceptualise and define the boundaries of area units for studying the determinants of health.

Findings showed that although census tracts may be homogeneous along independent indicators of active living potential, they were most often characterised by a combination of types of environment that were substantively different in terms of their active living potential. For this reason, census tracts should be used with caution as units of analysis when operationalising active living potential for studying determinants of walking. But census tracts or other administratively defined areas may be appropriate area units, i.e. may be homogeneous enough, when processes hypothesised to be operating on health are linked to the socioeconomic context of an area, for example affluence or poverty.

In this study, zones were delimited for methodological and aetiological purposes with the aim of minimising measurement errors of environmental characteristics and increasing internal validity of study design for measuring area effects on health. As can be expected, the zones are context-specific and cannot be exported to other geographic areas. Rather they are representations of the local realities of processes relating environmental characteristics to health. As suggested by others, the geographical aspects of the study design should be considered prior to conducting analyses [15]. Establishing the soundness of spatial units "of convenience" for representing the environmental and spatial processes under investigation should be part of the empirical approach for conceptualising, operationalising, and measuring area effects on health.

## Competing interests

The authors declare that they have no competing interests.

## Authors' contributions

MR conceptualised the study. She carried out spatial and statistical analyses, mapping of results, and drafted the manuscript. PA, LG and JMB participated in the conceptualisation the study, and in data analyses. All authors critically revised the paper, and read and approved the final manuscript.
